# [FeFe] hydrogenases with modified azadithiolate bridgeheads serve as unidirectional hydrogen oxidation catalysts

**DOI:** 10.1007/s00775-026-02141-4

**Published:** 2026-04-04

**Authors:** Manon T. Lachmann, James A. Birrell, Simone Morra, Patricia Rodríguez-Maciá

**Affiliations:** 1 School of Chemistry and Leicester Institute for Structural and Chemical Biology, University of Leicester, University Road, Leicester, LE1 7RH UK; 2 School of Life Sciences, University of Essex, Wivenhoe Park, Colchester, CO4 3SQ UK; 3 Faculty of Engineering, University of Nottingham, Coates Building, University Park, Nottingham, NG7 2RD UK

**Keywords:** [FeFe] hydrogenase, Active-site variant, Spectroscopy, Electrochemistry, Hydrogen oxidation, Proton transfer pathway

## Abstract

Semi-synthetic [FeFe] hydrogenases hold exciting potential as tuneable artificial metalloenzymes for efficient hydrogen conversion (H_2_ *⇌* 2 H^+^ + 2e^−^). This work investigates the role of the bridgehead ligand in the [FeFe] hydrogenase active site through the study of two bridgehead-modified active-site variants (2-oxapropane 1,3-dithiolate, ODT, and propane 1,3-dithiolate, PDT). Replacing the native 2-azapropane 1,3-dithiolate (ADT) bridgehead with ODT or PDT significantly modifies the active site’s electronic structure and disrupts proton-transfer; yet, some catalytic activity remains, suggesting that alternative catalytic pathways may exist. Notably, when adsorbed onto an electrode, the ODT enzymes operate as proficient catalysts for hydrogen oxidation at higher oxidative overpotentials, achieving current densities of up to 1 mA cm^− 2^, comparable to those observed for the respective ADT enzyme under the same conditions. Electrochemical analysis reveals similar catalytic behaviour between the ODT and PDT enzymes, suggesting that a proton transfer pathway between the active site and the protein environment still operates, albeit disrupted, in both bridgehead variants under these conditions. Insights into the electronic and catalytic interplay of these modifications could advance the optimisation of semi-synthetic [FeFe] hydrogenases and design of new bioinspired catalysts.

**Graphical abstract:**

**Supplementary Information:**

The online version contains supplementary material available at 10.1007/s00775-026-02141-4.

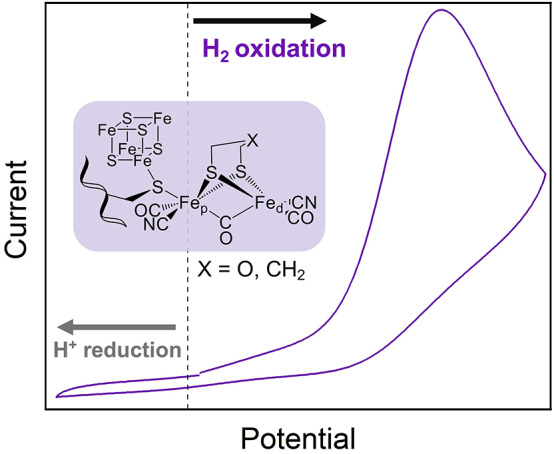

## Introduction

Dihydrogen (H_2_) serves as a valuable chemical feedstock for various industrial processes and, with its clean production and combustion, holds great promise as an energy vector for a renewable energy system [1–3]. Given this, the high demand for efficient, Earth-abundant catalysts for sustainable H_2_ production (2 H^+^ + 2e^−^ *\to* H_2_) and oxidation (H_2_ *\to* 2 H^+^ + 2e^−^) entails a significant scientific challenge. The search for new solutions has prompted scientists to investigate the potential of hydrogenase enzymes, which employ Fe and/or Ni active sites for efficient H_2_ conversion [4, 5]. Of the three types of hydrogenase enzymes, [FeFe] hydrogenases display the highest turnover rates and are the most efficient [6].

**Fig. 1:**
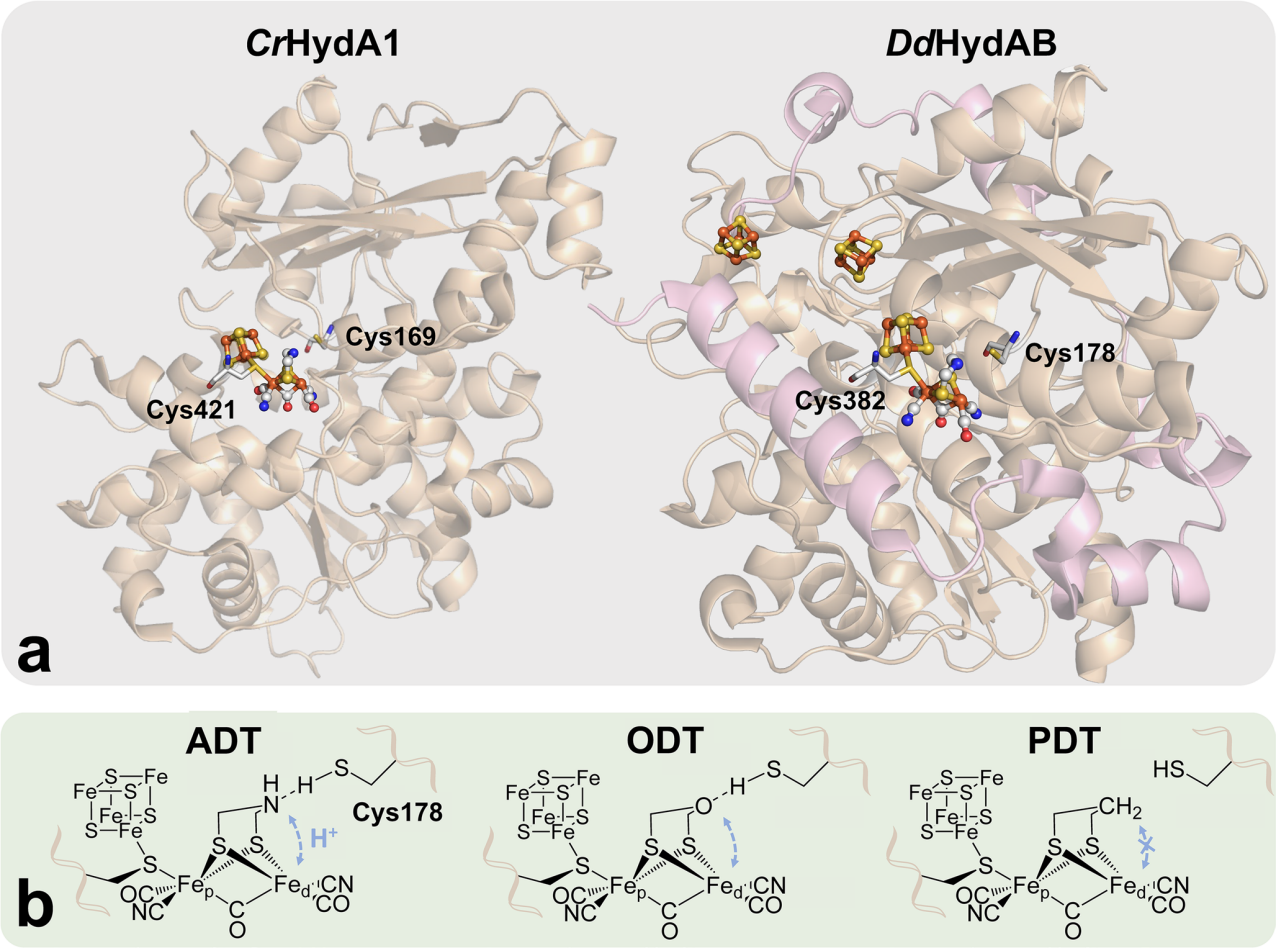
**a** Structure of [FeFe] hydrogenases *Cr*HydA1^ADT^ (PDB:6GM5) from *Chlamydomonas reinhardtii* and *Dd*HydAB^ADT^ (PDB:6SG2) from *Desulfovibrio desulfuricans* [16, 17]. HydA1 and HydA are shaded beige, HydB is pink. *Cr*HydA1^ADT^ has not yet been crystallised, and the figure displays the highest-resolution crystal structure of apo-*Cr*HydA1 reported, with a model of the [2Fe]_H_ cluster. **b** ChemDraw structures of the relevant H-clusters, including the native-like ADT bridging H-cluster and the propane 1,3-dithiolate (PDT) and 2-oxapropane 1,3-dithiolate (ODT) bridging H-cluster variants, are illustrated. The proposed interactions between the bridgehead and the surrounding protein environment are illustrated (using *Dd*HydAB nomenclature). The proton shuttle is highlighted in blue

The [FeFe] hydrogenase from *Desulfovibrio desulfuricans* (*Dd*HydAB) stands out among other hydrogenases for its exceptional activity and reversibility. Electrochemistry reveals activity at small deviations from the thermodynamic potential (i.e., negligible overpotential), and solution activity assays show turnover frequencies (TOFs) of up to 10,000 s^− 1^ for H_2_ production and 100,000 s^− 1^ for H_2_ oxidation, making it one of the most active hydrogenases known [7–9]. Nonetheless, this remarkable activity is offset by its extreme sensitivity to oxygen, which limits its practical application. *Dd*HydAB consists of two subunits, where the larger 44 kDa subunit (HydA) houses all the redox-active cofactors and the smaller 10 kDa subunit (HydB) wraps around HydA, providing structural support (Fig. 1a) [10]. The highly conserved active site comprises two subclusters: a diiron cluster [2Fe]_H_ and an iron-sulfur cubane cluster [4Fe-4 S]_H_, which are covalently linked through the sulfur of a cysteine residue (Cys382 in *D. desulfuricans* (Cys382_*Dd*_) Fig. 1a). The two Fe centres in [2Fe]_H_ are labelled distal (Fe_d_) and proximal (Fe_p_) based on their proximity to [4Fe-4S]_H_. Fe_d_ has a vacant coordination site in its apical position, where substrates (H_2_ and H^+^) and various inhibitors (CO, CN^−^, O_2_, etc.) bind [11–13]. Two additional [4Fe-4S] clusters (F-clusters) form an electron relay to facilitate electronic communication between the buried H-cluster and the protein surface [14]. These additional F-clusters complicate the redox chemistry of enzymes like *Dd*HydAB, presenting an extra challenge for understanding their catalytic mechanisms [15].

In 2013, Berggren and colleagues discovered an artificial maturation process, in which [FeFe] hydrogenase apoenzyme (containing [4Fe-4S]_H_ but lacking [2Fe]_H_ to complete the active site), recombinantly expressed in *Escherichia coli*, could be combined with a synthetically derived diiron active-site cofactor loaded onto a carrier protein (HydF) to yield a fully functional holoenzyme, with all necessary cofactors present [18]; soon after, it was demonstrated that the synthetic cofactor could be directly incorporated without HydF assistance [19]. This unlocked new research opportunities, allowing for the production and study of [FeFe] hydrogenase enzymes with synthetically modified active sites. The native-like 2-azapropane 1,3-dithiolate (ADT) bridgehead is depicted in Fig. 1b, alongside two active site variants featuring 2-oxapropane 1,3-dithiolate (ODT) and propane 1,3-dithiolate (PDT) bridgehead groups, in which the -NH group is replaced with a -CH_2_ or -O group [10, 20, 21]. This approach, combined with protein mutagenesis and advanced spectroscopic, structural, and computational studies, has enabled detailed research into the role of the unique ADT bridgehead group within the H-cluster and facilitated the isolation and extensive characterisation of numerous H-cluster states [22, 23]. The oxidised resting state prior to H_2_ binding or proton (H^+^) reduction is called H_ox,_ and it contains a mixed-valent (Fe_p_(II)Fe_d_(I)) [2Fe]_H_ cluster and an oxidised (2+) [4Fe-4S]_H_ cluster. One-electron reduction of H_ox_ yields a mixture of two states named here as H_red_ and H_red_H^+^ (but alternative nomenclature exists [11]). The H_red_ state has a reduced [4Fe-4S]_H_ cluster, while H_red_H^+^ is proposed to have a reduced and protonated [2Fe]_H_ cluster [24–26]. The site of protonation within the H_red_H^+^ state has yet to be experimentally verified. The one-electron reduced states can be further reduced to the H_sred_H^+^ state, where [4Fe-4S]_H_ is reduced (1+), and [2Fe]_H_ is reduced and protonated [27, 28]. The H_red_H^+^ and H_sred_H^+^ states can tautomerise to Fe-hydride-containing states named H_hyd:ox_ and H_hyd:red_, respectively, depending on whether [4Fe-4S]_H_ is oxidised or reduced [29]. Throughout this manuscript, we will refer to H_hyd:red_, with a reduced (1+) [4Fe-4S]_H_ and [Fe_d_(II)Fe_p_(II)]-H^−^ configuration, as H_hyd_ for simplicity. The catalytic cycle is believed to be completed upon protonation of H_hyd_, followed by H_2_ formation and release [22].

The ADT bridgehead group within the H-cluster is believed to play several essential roles in catalysis. Firstly, it likely forms a hydrogen bond with a nearby conserved cysteine residue (Cys178_*Dd*_), which is thought to be the terminal amino acid in the proton transfer pathway (PTP); it is widely accepted that this interaction is the site of proton exchange between the H-cluster and the protein environment and modulates the H-cluster’s electronic structure [11, 12, 16]. Secondly, its ability to act as a Lewis base facilitates proton-coupled electron transfer (PCET), which is believed to be crucial for activity. The ability to balance the increased negative charge on [2Fe]_H_ during electron transfer by protonating the ADT bridgehead promotes a low-energy reaction pathway [24, 26].

Previous research on [FeFe] hydrogenases with ODT bridgehead variants made use of the HydA1 [FeFe] hydrogenases from *Chlamydomonas reinhardtii* (*Cr*HydA1) and *Clostridium pasteurianum* (*Cp*HydA1) [11, 30–32]. Crucially, there are currently no published data on *Dd*HydAB^ODT^. The number of active-site states observed for the PDT and ODT semi-synthetic hydrogenases is far fewer than for ADT. [2Fe]_H_ of the PDT H-cluster can only be protonated at Fe_d_ and, therefore, cannot form H_red_H^+^ or H_sred_H^+^ but has been observed in H_ox_, H_red_ and H_hyd_ states [15, 33]. Two additional H-cluster states (named HoxH and Hred’H) have been observed in which the H_ox_ and H_red_ states are proposed to be protonated at [4Fe-4S]_H_ [34]. However, recently, data suggest an alternative explanation for these states [35]. In *Cr*HydA1^ODT^, the H_hyd_ state was found to be especially stable, and the enzyme was isolated in this state after maturation [31]. Along with H_hyd_, *Cr*HydA1^ODT^ was also observed in H_ox_ and H_red_ states [11, 30].

*Cp*HydA1^PDT^ and *Cp*HydA1^ODT^ have been crystallised in their resting state and shown to exhibit negligible catalytic activity [18, 32]. Siebel and coworkers observed residual activity in *Cr*HydA1^PDT^ and proposed that, because the bridgehead cannot be protonated, a different catalytic mechanism must be operative [36]. They hypothesised that protonation might occur on one of the sulfurs of the dithiolate bridge. In a synthetic analogue of the [FeFe] hydrogenase active site, ODT bridges were shown to facilitate proton transfer to and from Fe_d_, although its proton-transfer capability was limited to strongly acidic conditions due to ODT’s weak basicity [37]. Typically, the pK_a_ values of ethers are reported in the range of 40–50, indicating a very low propensity for protonation, compared with dimethylamine, which has a pK_a_ value of 10.73 [38–40]. This likely explains why ODT exhibits catalytic activity similar to PDT rather than ADT. Nonetheless, we believed this required further investigation using different [FeFe] hydrogenases, as numerous studies have demonstrated the significant influence of the protein environment on the H-cluster’s characteristics and catalytic function [15, 23, 41–43]. We chose *Cr*HydA1 to corroborate our findings with existing research and *Dd*HydAB, which contains additional F-clusters, to examine how they influenced the behaviour of the modified active sites. Given the extensive spectroscopy already reported for *Cr*HydA1^PDT^ and *Dd*HydAB^PDT^, this study will mainly focus on their catalytic properties, while additional spectroscopic characterisation of *Cr*HydA1^ODT^ and *Dd*HydAB^ODT^ has been conducted to support our functional analysis.

## Results and discussion

### Functional characterisation of *Cr*HydA1^PDT^, *Cr*HydA1^ODT^, *Dd*HydAB^PDT^and *Dd*HydAB^ODT^

To investigate the catalytic properties of *Dd*HydAB and *Cr*HydA1 maturated with PDT- and ODT-modified active sites, solution activity assays and protein film electrochemistry (PFE) were performed. Table 1 displays the H_2_ oxidation and H_2_ production turnover frequencies (TOFs) of *Dd*HydAB and *Cr*HydA1 maturated with ADT, ODT, and PDT. All reported H_2_ oxidation activities were determined in this work from solution assays with benzyl viologen (BV) as an electron acceptor (details of the experimental procedure are provided in the SI, cf. Fig. S1 for bar chart representation of the TOFs). H_2_ production assays have previously been reported for *Dd*HydAB^ADT^, *Cr*HydA1^ADT^, *Cr*HydA1^ODT,^ and *Cr*HydA1^PDT^ (Table 1). H_2_ production assays performed on *Dd*HydAB^ODT^ in this study exhibited undetectable activity. Previous publications reporting ODT enzymes as inactive reported only H_2_-evolution activity; our data demonstrate that both ODT and PDT enzymes exhibit some H_2_-oxidation activity, with ODT being more active than PDT. *Dd*HydAB achieves considerably higher activities than *Cr*HydA1, with every active site in *Dd*HydAB outperforming its *Cr*HydA1 counterpart. This has already been established for the native ADT enzymes [7, 44], but here we show the same trend for ODT and PDT. However, the activity differences between the respective *Dd*HydAB and *Cr*HydA1 enzymes are varied: *Dd*HydAB^ADT^ is approximately 400-fold more active than *Cr*HydA1^ADT^, while *Dd*HydAB^ODT^ is 10-fold and *Dd*HydAB^PDT^ 100-fold more active than their *Cr*HydA1 counterparts.

**Table 1:** Turnover frequencies (TOFs) of the semi-synthetic [FeFe] hydrogenases *Cr*HydA1 and *Dd*HydAB maturated with ADT, ODT and PDT H-clusters. The limit of detection (LOD) for the H_2_ production assay in this work was 0.10 s^− 1^

[FeFe] hydrogenase	H-cluster	Turnover frequency (s^− 1^)		
		H_2_ oxidation	H_2_ production	
			Value	Ref.
*Dd*HydAB	ADT	77,000 ± 2100	3700 ± 400	[45]
	ODT	10 ± 0.24	< LOD	This work
	PDT	1.2 ± 0.24	Not determined	-
*Cr*HydA1	ADT	190 ± 18	250 ± 32	[19]
	ODT	0.98 ± 0.16	Not detectable	[18]
	PDT	0.015 ± 0.0035	0.76 ± 0.25	[36]

**Fig. 2:**
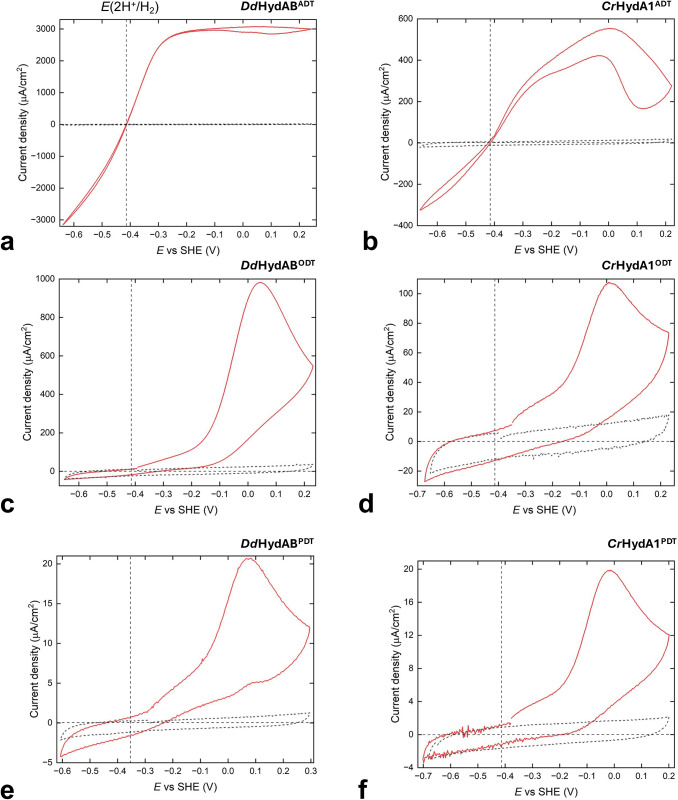
Cyclic Voltammograms (CVs) of *Dd*HydAB^ADT^ (**a**), *Cr*HydA1^ADT^ (**b**), *Dd*HydAB^ODT^ (**c**), *Cr*HydA1^ODT^ (**d**), *Dd*HydAB^PDT^ (**e**), and *Cr*HydA1^PDT^ (**f**). The electrode blank (grey dashed line) is displayed. The vertical dashed line corresponds to *E*(2H^+^/H_2_) (at pH 7 is -0.413 V vs. SHE and at pH 6 is -0.355 V vs. SHE), and the dashed horizontal line corresponds to the zero current density. Measurements were performed in buffer mix pH 6 (e) or 7 (a-d, f), 0.1 V s^− 1^ scan rate, 3300 rpm, 1000 mL min^− 1^ H_2_ and at 25 °C

Protein film electrochemistry (PFE) was performed to gain a better understanding of the catalytic behaviours of each variant. Figure 2 displays the cyclic voltammograms (CVs) of *Dd*HydAB and *Cr*HydA1 maturated with ADT, ODT, and PDT. The CVs of both ADT enzymes (Fig. 2a and b) are similar to those previously reported, with *Dd*HydAB^ADT^ and *Cr*HydA1^ADT^ exhibiting reversible catalysis characterised by low overpotential and high electrocatalytic current densities, with *Dd*HydAB^ADT^ achieving 5–10 fold higher current densities [45–49]. The lower electrocatalytic current densities observed with *Cr*HydA1^ADT^ compared to *Dd*HydAB^ADT^ align with our quantitative results (Table 1). However, these data are not directly comparable, as we cannot determine the hydrogenase surface coverage on the electrode and therefore cannot obtain quantitative results. This is true for direct comparison between *Dd*HydAB and *Cr*HydA1, which differ in size and surface charge. However, bridgehead modification to the H-cluster is unlikely to influence protein adsorption onto the electrode, since the protein’s size and surface charge distribution are not expected to change. Thus, comparing the CVs of different H-clusters within the same hydrogenase should provide some insight into the redox behaviour of the modified active sites.

*Dd*HydAB^ODT^ and *Cr*HydA1^ODT^ (Fig. 2c and d) show substantial electrocatalytic current densities only in the H_2_ oxidation direction, in agreement with solution assays, and reveal that, at large overpotentials, considerable electrocatalytic current is achieved. *Dd*HydAB^ODT^ retains around a third, and *Cr*HydA1^ODT^ a fifth of the maximum current densities achieved by their ADT counterparts. Identical experiments performed in the absence of H_2_ confirmed that the observed electrocatalytic currents were due to H_2_ oxidation activity (Fig. S2a and S2b) and demonstrated that the ODT and PDT variants are inactive for H_2_ evolution, as no electrocatalytic current was observed even in the absence of H_2_, which has been shown to weakly inhibit H_2_ evolution in native (ADT) [FeFe] hydrogenases [50]. Although the maximum current densities achieved by the PDT enzymes (Fig. 2e and f) remain modest (20 µA/ cm^2^), a similar increase in current density at large oxidative overpotential is observed, as with ODT. In fact, the catalytic profiles of *Dd*HydAB^ODT^, *Cr*HydA1^ODT^, *Dd*HydAB^PDT^ and *Cr*HydA1^PDT^ are very similar and clearly differ from both ADT enzymes, so we can hypothesise that, mechanistically, ODT behaves more like PDT than ADT. Kisgeropoulos and colleagues previously demonstrated that disrupting the PTP (in their case, with a C169S_*Cr*_ mutation) impairs proton reduction more severely than oxidation [51]. This would imply that the PTP has been disrupted in the ODT and PDT variants.

In contrast to the observations from PFE, in solution assays, both ODT and PDT enzymes exhibited significantly poorer performance, displaying relatively insignificant activity compared to their ADT counterparts (Table 1). This discrepancy likely arises because, in solution assays, activity is measured near the thermodynamic potential of H_2_ oxidation (assuming a BV solution potential around − 300 mV vs. SHE at pH 7 [52] vs. *E*(2H^+^/H_2_) of -413 mV at pH 7) and at such low overpotentials ( ≈ + 100 mV), the activity differences between ADT, ODT and PDT observed in PFE (Fig. 2) agree with the solution assays.

Another interesting observation is that while *Dd*HydAB^ADT^ and *Cr*HydA1^ADT^ show reversible loss of H_2_ oxidation electrocatalytic current at high potential (so-called high-potential inactivation, HPI [9]), the ODT and PDT enzymes display irreversible current loss. While some degree of film loss occurs for *Cr*HydA1 (as shown in Fig. S3b, where *Cr*HydA1^ADT^ loses ∼ 33% of H_2_ production current between the first and second CVs and ∼ 27% between the second and third), contributing to the gradual decrease in current density after each scan, it does not fully account for the ∼ 62% and ∼ 68% current losses observed during H_2_ oxidation by *Cr*HydA1^ODT^ and *Cr*HydA1^PDT^ (Fig. S3d and S3f), respectively. Meanwhile, *Dd*HydAB^ADT^ exhibits essentially no current loss in subsequent CVs (Fig. S3a), whereas *Dd*HydAB^ODT^ and *Dd*HydAB^PDT^ (Fig. S3c and S3e) display the same current loss behaviour as their *Cr*HydA1 counterparts. Previous work by del Barrio and colleagues proposed that halide ions coordinating with a protonated form of the amine in the bridgehead and Cys178_*Dd*_/Cys169_*Cr*_ are responsible for HPI [53]. This cannot account for the HPI observed in both *Dd*HydAB^PDT^ and *Cr*HydA1^PDT^, where there is no potential to form a halide adduct to the bridgehead. The irreversible nature of HPI in the ODT and PDT variants may suggest that the inactivation mechanism differs from that observed in ADT, where it is reversible. However, the comparable onset potentials of HPI observed across ADT, ODT, and PDT may suggest that aspects of this inactivation process are similar in all six enzymes.

### Spectroscopic Characterisation of *Cr*HydA1^ODT^ and *Dd*HydAB^ODT^

**Fig. 3:**
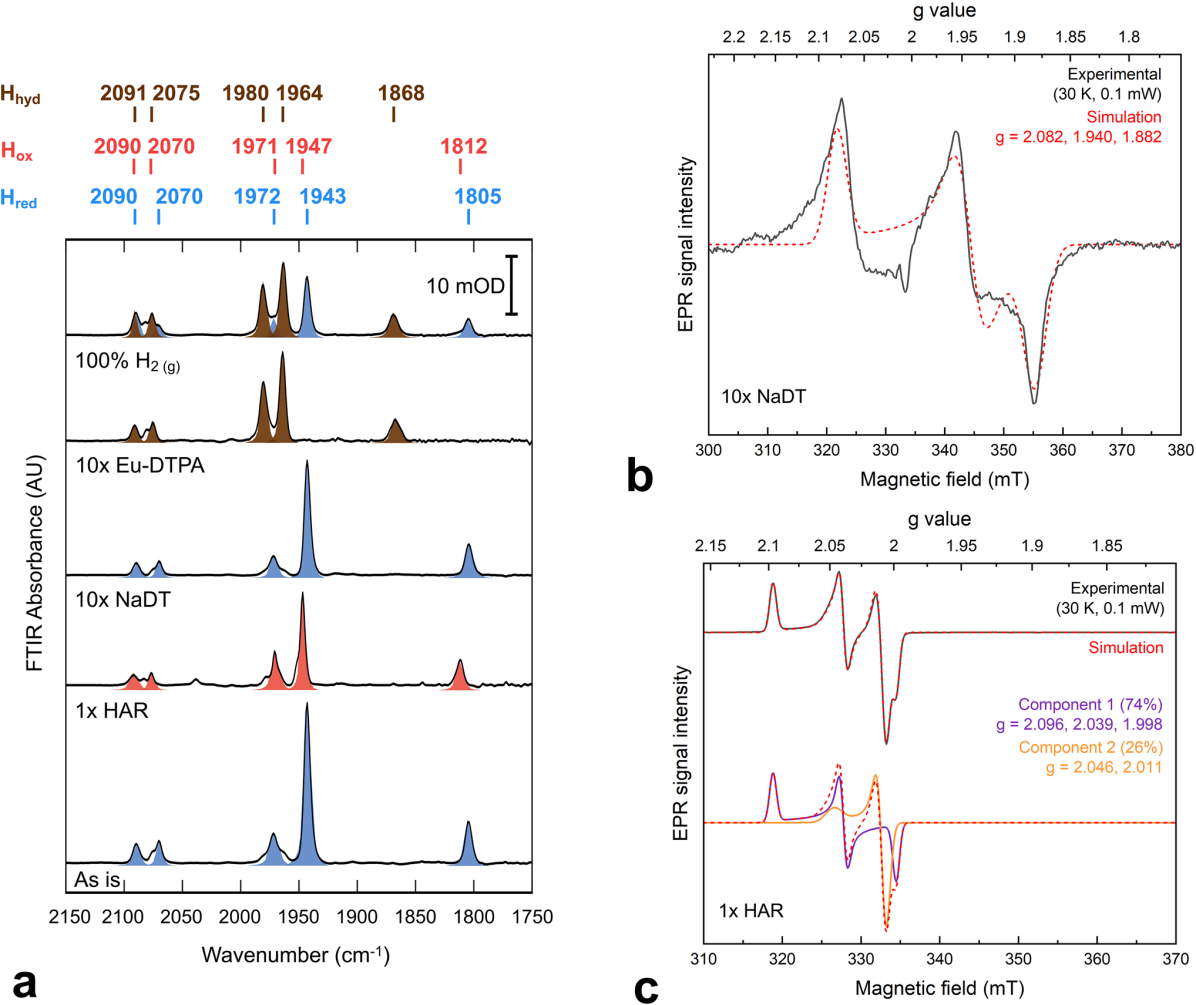
**a** FTIR spectra of 2mM *Cr*HydA1^ODT^ under various conditions (listed underneath each spectrum). The ‘as isolated’ (As is) spectrum corresponds to untreated *Cr*HydA1^ODT^ measured directly after maturation. Alongside are EPR spectra of 200 µM *Cr*HydA1^ODT^ treated with 10x NaDT (**b**) and 1x HAR (**c**). The experimental (black) and simulated (red dashed) spectra are overlaid. In (**c**), the simulated spectrum is separated into its different components below. The percentages alongside the components represent their contributions to the sample based on the relative numbers of spins calculated from the double integrals of the spectra (assuming no H_red_ was present in the sample)

We were interested to see whether the spectroscopic properties of *Cr*HydA1^ODT^, *Cr*HydA1^PDT^, *Dd*HydAB^ODT,^ and *Dd*HydAB^PDT^ would align with the similar catalytic behaviour observed across the ODT and PDT variants. Spectroscopic characterisation of ‘as-isolated’ (as is) *Cr*HydA1^ODT^ (Fig. 3a) indicates that it is likely in the H_red_ state after maturation (under 3% H_2_). Its EPR spectrum agrees well with the enzyme being predominantly in the H_red_ state, as it shows very little signal intensity, characteristic of a singly reduced EPR-silent H-cluster comprising antiferromagnetically coupled Fe_p_(II)Fe_d_(I) and reduced (1+) [4Fe-4S]_H_ subclusters. The small amount of signal observed can be simulated by a mixture of species corresponding to H_ox_, H_ox_-CO, and H_hyd_ states (see Fig. S4 for a simulation). Reduction of *Cr*HydA1^ODT^ with sodium dithionite (NaDT, *E°* = -0.66 V vs. SHE [54]) generated an unchanged IR spectrum. Reduction with Eu(II)-diethylenetriamine (Eu(II)-DTPA, *E°* = -1.14 V vs. SHE [55]) generated a spectrum exhibiting pure H_hyd_, previously characterised with Fe_p_(II)Fe_d_(II)-H^−^ and reduced (1+) [4Fe-4 S]_H_ subclusters [11, 30, 31]. EPR analysis of the NaDT-reduced state (Fig. 3b) revealed a spectrum characteristic of the H_hyd_ state. Based on the number of spins, it was inferred that the sample comprised 96% (EPR-silent) H_red_ and 4% H_hyd_. Flushing *Cr*HydA1^ODT^ (as is) with 100% H_2_ for 10 min resulted in a mixture of H_hyd_ and H_red_ states. Comparing the intensity of the 1943 cm^− 1^ peak for the as-isolated and H_2_-flushed spectra, there was 64% conversion to H_hyd_. Oxidation with 2 mM hexaamineruthenium(III) chloride (HAR, *E* ^*O*^ = + 0.05 V vs. SHE [56]) produced a state in which the CO and CN^−^ stretching frequencies were shifted by ∼ 3 cm^− 1^ to higher wavenumbers, consistent with the oxidation of [4Fe-4 S]_H_ [15]. Its EPR spectrum (Fig. 3c) exhibits sharp, distinct signals which are characteristic of H_ox_ (component 1), where the H-cluster comprises Fe_p_(II)Fe_d_(I) and oxidised (2+) [4Fe-4 S]_H_ subclusters [57, 58]. The spectrum contains an additional component characteristic of the H_ox_-CO state, indicating that some degradation of the H-cluster (leading to CO release and uptake by active enzymes) occurred during sample freezing [21].

*Cr*HydA1^ODT^ has been well characterised in previous studies [11, 30, 31]. Generally, our characterisation aligns well with previous data. Duan and colleagues found that maturation of *Cr*HydA1^ODT^ produced a mixture of H_red_ and H_hyd_ states [11]. They demonstrated H_ox_ formation via autooxidation using a humidified N_2_ gas stream, and reformation of H_red_ and H_hyd_ by introducing H_2_ into the stream. We have demonstrated that these states can also be formed through chemical oxidation and reduction. Interestingly, in these investigations, a significant proportion of *Cr*HydA1^ODT^ was isolated in the H_hyd_ state after maturation (the rest in H_red_) [11, 30, 31]. Compared with our method, the main difference was that the maturation in Duan et al. was performed using 2 mM NaDT. When we repeated maturation with NaDT, we obtained a mixture of H_hyd_ and H_red_ states (Fig. S5a). However, when NaDT was added after maturation, H_hyd_ formation did not occur (Fig. 3a). A potential explanation for this is that since the [2Fe]_H_ subcluster within the synthetic ODT cofactor exists in Fe(I)Fe(I) redox states and the [4Fe-4S]_H_ subcluster in the apo-enzyme is reduced (to + 1) in the presence of NaDT, during maturation, the system forms a highly reactive state (with Fe(I)Fe(I) at [2Fe]_H_) that immediately acquires a proton to generate the H_hyd_ state (with Fe(II)Fe(II) at [2Fe]_H_). Maturation without NaDT, where the [4Fe-4S]_H_ cluster is in the oxidised (+ 2) state, favours formation of H_red_ by transfer of an electron from [2Fe]_H_ to [4Fe-4S]_H_ prior to hydride formation. Subsequent addition of NaDT cannot reduce [2Fe]_H_, and a stronger reducing agent, namely Eu(II)-DTPA, is required for H_hyd_ formation. The presence of H_hyd_ in small proportions in both the as-isolated and NaDT reduced samples (Fig. 3b and S4) of *Cr*HydA1^ODT^ is likely due to the 3% H_2_ atmosphere of the glovebox.

**Fig. 4:**
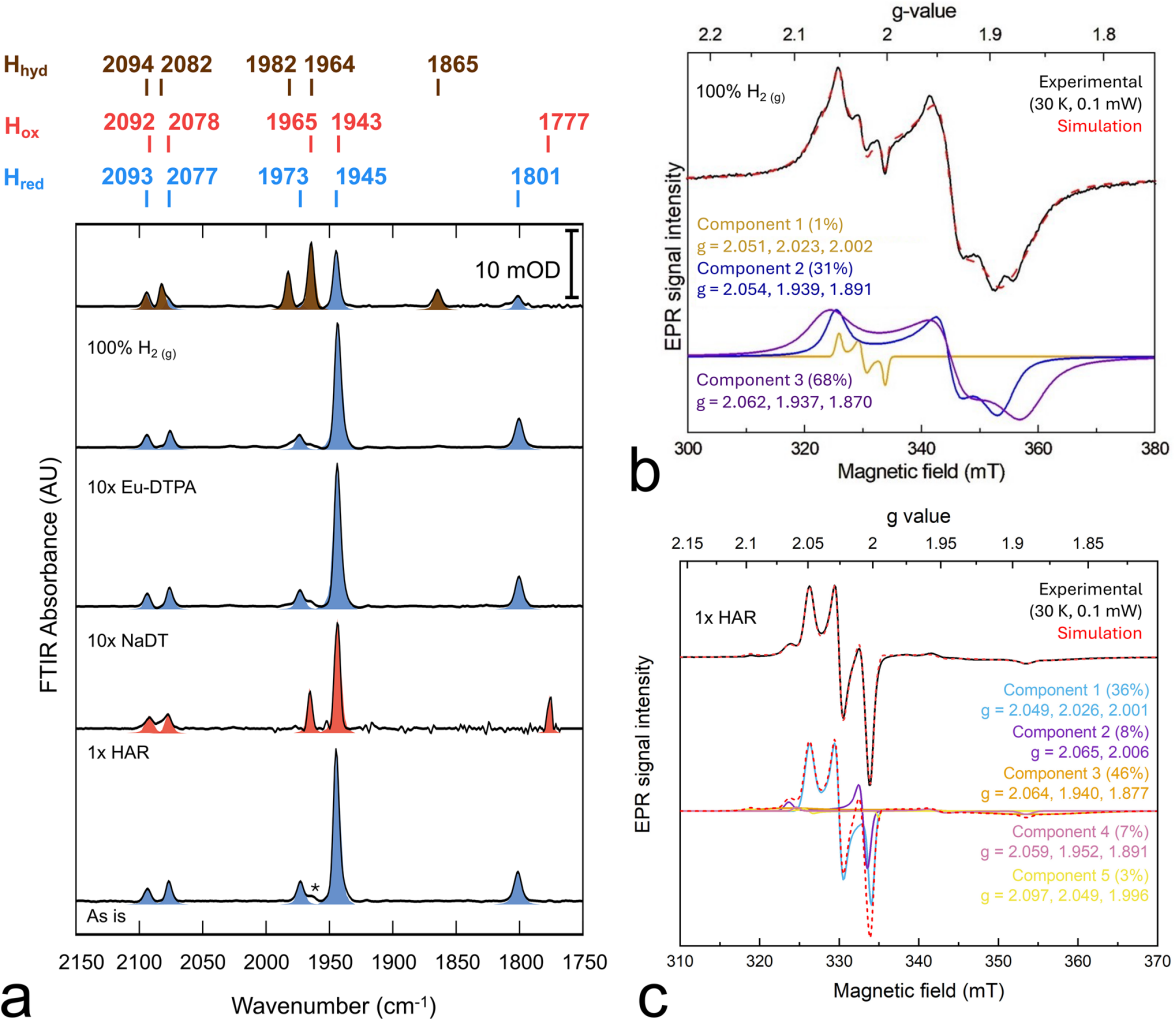
**a** FTIR spectra of 1 mM *Dd*HydAB^ODT^ under various conditions (listed underneath each spectrum). The asterisk above the as is spectrum denotes a peak at 1982 cm^− 1^. **b** EPR spectrum of 200 µM *Dd*HydAB^ODT^ flushed with 100% H_2_ for 10 min. The experimental (black) and simulated (red dashed) spectra are overlaid; below, the simulated spectrum is separated into its different components. In **c**, the experimental (black) and simulated (red dashed) spectra of *Dd*HydAB^ODT^ treated with 1x HAR are overlaid; below, the simulated spectrum is separated into its different components. The percentages alongside the components represent their respective contributions to the sample based on their relative number of spins calculated from the double integrals of the spectra

The same experiments were performed with *Dd*HydAB^ODT^ (Fig. 4). Like *Cr*HydA1^ODT^, *Dd*HydAB^ODT^ also exists in the H_red_ state after maturation (under 3% H_2_); the broad weak EPR signals observed (Fig. S6) are characteristic of an EPR-silent H-cluster and spin-coupled reduced F-clusters [21]. While its IR spectrum resembles that of the H_red_ state of *Cr*HydA1^ODT^, the stretching frequencies of the CN^−^ and CO ligands generally appear at higher wavenumbers in *Dd*HydAB^ODT^, a finding also observed in the ADT and PDT enzymes, and is likely due to the subtle differences in the active-site environment. Reduction with both NaDT and Eu(II)-DTPA did not affect *Dd*HydAB^ODT^, as indicated by unchanged IR spectra. However, flushing the sample with 100% H_2_ for 10 min produced an IR spectrum containing peaks similar to those of the H_hyd_ state in *Cr*HydA1^ODT^ with additional peaks attributed to H_red_. Its EPR spectrum (Fig. 4b) supports the IR interpretation, with a large contribution (component 3) characteristic of the H_hyd_ state [31], a smaller contribution (component 2) characteristic of H_red_ with the signal attributed to reduced F-clusters, and a negligible contribution from H_ox_ (component 1). Comparing the intensity of the 1945 cm^− 1^ peak between the as-isolated and H_2_-flushed spectra, there is approximately 66% conversion to H_hyd_, aligning well with our EPR interpretation. The peak at 1982 cm^− 1^ in the as-isolated spectrum (Fig. 4a) implies some H_hyd_ may be present after maturation, but no other peaks attributed to H_hyd_ are observed. If it is present at a low proportion, as with *Cr*HydA1^ODT^, it is likely due to the 3% H_2_ atmosphere in our glovebox. Upon oxidation with 1 mM HAR, the IR peaks unexpectedly shifted to lower frequencies (Fig. 4a) rather than higher frequencies as observed for *Cr*HydA1(Fig. 3a). However, EPR measurements (Fig. 4c) confirm that treatment with 2 mM HAR yields the H_ox_ state (component 1), suggesting that [4Fe-4S]_H_ has been oxidised [57]. The EPR spectrum contains multiple additional components; component 2 is typical of the H_ox_-CO state, indicating that, as with *Cr*HydA1^ODT^, the H-cluster was partially degraded upon sample freezing and components 3 and 4 display signals characteristic of reduced spin-coupled F-clusters [21]. The identity of component 5 (contributing just 3%) remains unknown. As previously mentioned, the two additional F-clusters within *Dd*HydAB complicate its redox chemistry and make spectroscopic characterisation more complex. Here, EPR data are particularly challenging to interpret compared with those from *Cr*HydA1^ODT^, requiring more components to accurately simulate the data. Table S1 defines the states of *Dd*HydAB that can be observed via EPR, including all three redox centres.

The IR spectrum of *Dd*HydAB^ODT^ in the H_ox_ state was surprising because oxidation typically results in blue-shifted (towards higher frequencies) CO and CN^−^ stretching frequencies relative to H_red_, as the Fe centres become less electron-rich and thus engage in weaker backbonding. However, in this case, the stretching frequencies were red-shifted (towards lower frequencies), suggesting either increased back-donation from the Fe centres (i.e., reduction) or a change in the interactions between the H-cluster and the protein environment. As EPR suggests oxidation of [2Fe]_H_ yielding an H_ox_-like spectrum, it seems likely that a structural change to the protein or a conformational change to the H-cluster occurs upon oxidation, causing the IR bands to red-shift relative to H_red_.

**Fig. 5:**
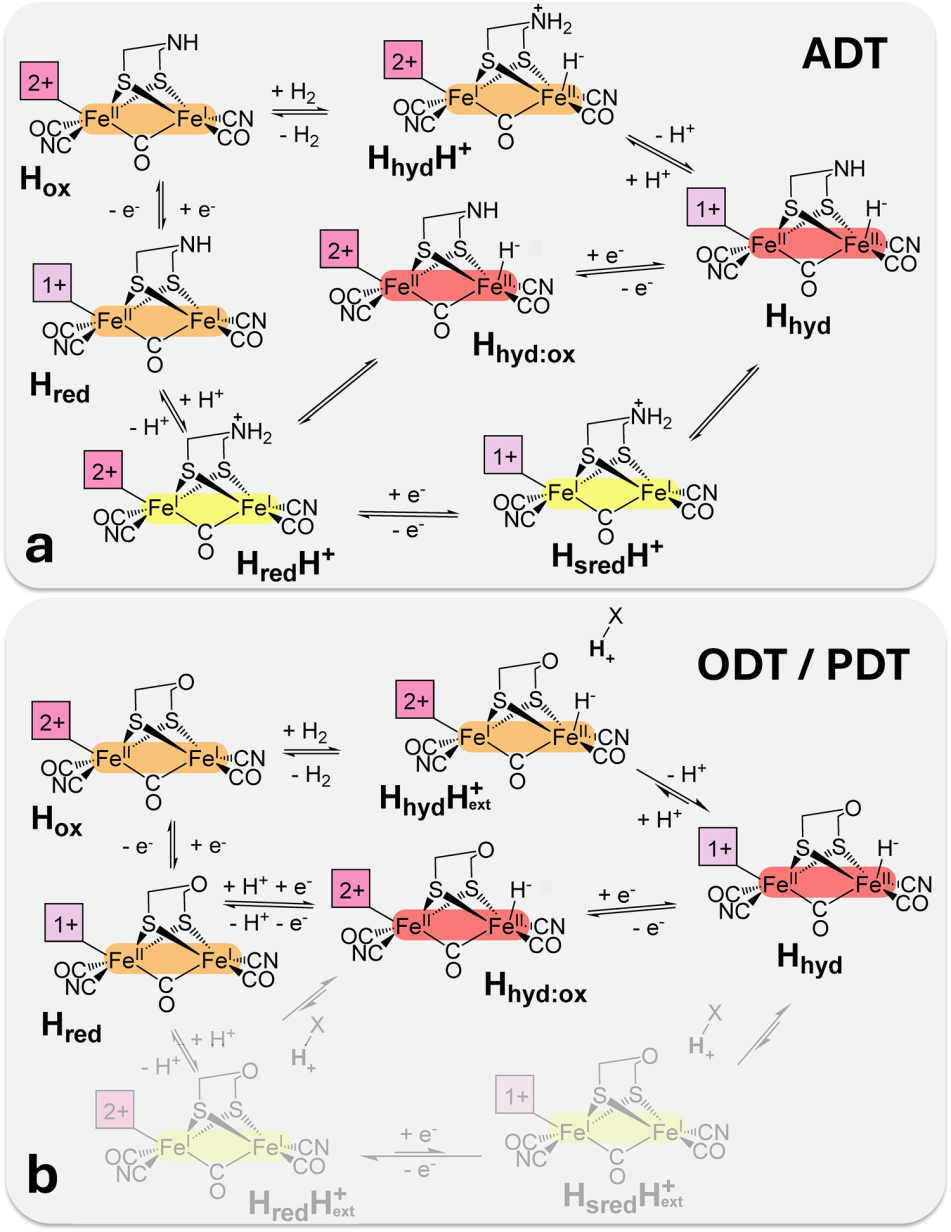
Simplified representations of the H-cluster showing proposed H_2_ oxidation pathways for ADT (**a**) and ODT and PDT (**b**). In (**b**), X denotes the species responsible for proton transfer between Fe_d_ and the protein environment; H_ext_ denotes a protonation event external to the H-cluster. Different colours represent the various oxidation states of [4Fe-4S]_H_ and [2Fe]_H_. PCET steps are: H_red_ *⇌* H_red_H^+^ and H_hyd_H^+^ *⇌* H_hyd_

Another interesting observation from these experiments was that hydride formation in *Dd*HydAB^ODT^ required H_2_ as a substrate, whereas in *Cr*HydA1^ODT^ it also occurred via chemical reduction. We may be able to explain the formation of H_hyd_ after incubation with H_2_ by referring to the catalytic mechanism. Figure 5 outlines the proposed catalytic pathways for H_2_ oxidation in the native ADT (Fig. 5a) and the ODT and PDT (Fig. 5b) enzymes. H_2_ oxidation can proceed through two pathways; one involves the formation of a two-electron-reduced (super-reduced) H_sred_H^+^ state, and the other bypasses the super-reduced state by forming an Fe-hydride intermediate with an oxidised (2+) [4Fe-4S]_H_ cluster (H_hyd:ox_). Under a constant flow of 100% H_2_, [4Fe-4S]_H_ will likely remain reduced, preventing the formation of H_hyd:ox_. The -CH_2_- group of the PDT bridgehead cannot undergo protonation, and given ODT’s similar catalytic behaviour (Fig. 2) and its high pK_a_ [37], it seems likely that the -O- group cannot either. If the ODT H-clusters can only be protonated at Fe_d_, then forming H_sred_H^+^ would also be prevented, as the increased negative charge on the super-reduced state can no longer be balanced by the second protonation of [2Fe]_H_. This would mean a high activation barrier for its formation, and *Dd*HydAB^ODT^ and *Cr*HydA1^ODT^ would therefore be kinetically trapped in the H_hyd_ state. In order to generate the H_hyd_ state via chemical reduction, an electron must be transferred from the reductant to [2Fe]_H_ with concomitant proton transfer from the PTP. Both processes will be slow, particularly with NaDT, and may be outcompeted by the subsequent formation and release of H_2_, thereby preventing the buildup of the H_hyd_ intermediate. Use of a very low-potential reductant (Eu(II)-DTPA) enables more rapid electron transfer, yielding a larger amount of H_hyd_ in *Cr*HydA1. The problem with *Dd*HydAB is that the active site is much more buried than in *Cr*HydA1, and electron transfer must proceed via the F-clusters, whose redox potentials are not sufficiently negative for rapid electron transfer to [2Fe]_H_. Attempts to form H_hyd_ in *Dd*HydAB^ODT^ during maturation through the addition of NaDT yielded small amounts of H_hyd_ (Fig. S5b). Its presence could be attributed to the 3% H_2_ in the glovebox atmosphere. Our earlier rationale for H_hyd_ formation in *Cr*HydA1^ODT^ after maturation with NaDT rested on the availability of a nearby proton source, though we did not speculate on its identity. If this is true, the proton source may be unavailable in *Dd*HydAB, preventing the formation of H_hyd_. However, an alternative explanation may involve the F-clusters present in *Dd*HydAB^ODT^. During maturation of the H-cluster with NaDT, the initially formed [4Fe-4S]_H_^+^-[Fe(I)Fe(I)] can either undergo oxidation, yielding a [4Fe-4S]_H_^+^-[Fe(II)Fe(I)] state (H_red_), or undergo protonation to yield a [4Fe-4S]_H_^+^-[Fe(II)Fe(II)]-H^−^ state (H_hyd_). We suggest that for maturation of *Cr*HydA1^ODT^ in the presence of NaDT, the protonation route is favoured, while for *Dd*HydAB^ODT^, the oxidation route is favoured. However, further investigation is needed.

This hypothesis may also help explain the catalytic profiles we observe for both ODT and PDT variants (Fig. 2b, c, d and e). PFE showed that the ODT and PDT variants were active for H_2_ oxidation, but their activities were very low at small oxidative overpotentials, with large overpotentials required to reach full activity. At low oxidative potentials, we observed poor electrocatalytic currents and turnover frequencies, consistent with the H_sred_H^+^ catalytic pathway dominating; this would be slow for the same reason that we trap H_hyd_ when the ODT variants are flushed with H_2_. At higher overpotentials, the H_hyd: ox_ pathway dominates, bypassing the high-energy H_sred_H^+^ intermediate and greatly enhancing the activity of the ODT and PDT variants. This mechanism (Fig. 5b) implies that the ODT and PDT variants may transiently form one-electron-reduced [2Fe]_H_ states without concomitant protonation of [2Fe]_H_. This has been observed previously in the sensory hydrogenase from *Thermotoga maritima* (*Tm*HydS) [59]. The differences in activity between ODT and PDT could then be explained by the inability to form the protonated H_red_H^+^ and H_sred_H^+^ states. If instead, the catalytic mechanism requires the unprotonated forms to be transiently adopted, this will slow down catalysis during proton reduction (as forming the Fe(I)Fe(I) intermediate is slow) and yield a very high overpotential during H_2_ oxidation as the potential of H_hyd:ox_/H_ox_ is very positive ([2Fe]_H_ is in an Fe(II)Fe(II) state). We would expect the hydrogen bond formed between the ADT and Cys178_*Dd*_/Cys169_*Cr*_ residues to be conserved in ODT. This interaction may enhance the activity of ODT relative to PDT, for example, by stabilising H-cluster intermediates or facilitating proton transfer. However, it is likely that, in both variants, the disrupted PTP cannot function as effectively without the bridgehead’s involvement, and thus, rates comparable to those of their ADT counterparts cannot be achieved.

Another implication of this hypothesis is that the disrupted PTP transfers protons directly to Fe_d_. Whether proton transfer to Fe_d_ occurs directly from the terminal amino acid in the normal PTP (i.e., Cys178_*Dd*_/169_*Cr*_) or from some other nearby amino acid residue remains unclear, but the similar catalytic profiles suggest that this mechanism is conserved between *Dd*HydAB and *Cr*HydA1. Given the considerable current densities achieved by the ODT enzymes compared with their ADT counterparts, this alternative proton transfer can function efficiently at sufficiently high overpotentials.

Similar catalytic profiles to those of the ODT and PDT variants have been observed in the E28Q variant of the Hyd1 [NiFe] hydrogenase from *E. coli*, which exhibits a strong catalytic bias toward H_2_ oxidation [60]. Here, a conserved glutamate, crucial in the PTP, was substituted for glutamine to further examine its specific role in catalysis. When the E28Q variant was analysed by PFE, a catalytic profile was observed, characterised by a period of low activity at small oxidative overpotentials, followed by significant activation at large overpotentials, similar to the catalytic profiles observed for the ODT and PDT variants (Fig. 2). It was demonstrated here that this activation was due to a hydroxide ion that presented at large oxidative overpotentials and served in place of the glutamate to restore the PTP. A similar mechanism may be at play here, with a hydroxide ion presenting at high oxidative overpotential and acting in place of the non-functional ODT and PDT bridgeheads to accelerate proton transfer and enhance activity. However, the crystal structure of *Cp*HydA1^ODT^, does not show any additional water molecules compared with the ADT enzyme [32]. A similar activation was also observed in the E144D and E141D variants of *Cr*HydA1^ADT^ [61]. These glutamic acid residues make up part of the PTP in *Cr*HydA1 and were studied to determine the extent to which catalysis depends on long-range coupling between electron and proton transfer. These variants exhibited similar electrocatalytic profiles characterised by a period of low activity followed by activation at large oxidative overpotential. These instances provide precedent for restoration of the PTP in ODT and PDT variants as a reason for the observed activation at large oxidative overpotential.

## Conclusion

In this work, we have shown that *Dd*HydAB^ODT^ and *Cr*HydA1^ODT^ exhibit electrocatalytic behaviour similar to that of their PDT counterparts, which is notably different from the native ADT hydrogenases. This was corroborated by spectroscopic characterisation, which showed that, in most cases, the enzymes were isolated in the same states under similar conditions. The discrepancies we observed primarily involved *Dd*HydAB^ODT^, including its inability to form H_hyd_ during chemical reduction and the red-shift of the CO and CN^−^ stretching frequencies in H_ox_ relative to H_red_. The reasons for these spectroscopic discrepancies are not yet fully understood and remain the focus of ongoing work. Despite this, it maintained a similar catalytic profile to *Cr*HydA1^ODT^, *Dd*HydAB^PDT,^ and *Cr*HydA1^PDT^, suggesting these discrepancies are not important to catalysis. This provides evidence that the catalytic mechanisms in ODT and PDT are similar, that, like PDT, ODT cannot be protonated, and that proton transfer between the H-cluster and the protein environment is disrupted.

Replacing the ADT bridgehead with ODT or PDT completely deactivates H_2_ evolution and severely hinders H_2_ oxidation at low oxidative overpotential. However, at large oxidative overpotential, the ODT and PDT enzymes are activated. Solution assays were unable to detect this activation - an occurrence previously reported [62], underscoring the importance of PFE as a functional technique. We hypothesise that the activation we observe at large overpotential may be due to two reasons: *(1)* proton transfer is partially restored, as similarly observed in [NiFe] and [FeFe] hydrogenase variants with disrupted PTPs [60, 61], and *(2)* a shift in the catalytic pathway towards H_hyd:ox_ formation, bypassing H_sred_H^+^ formation. The latter would be consistent with [2Fe]_H_ being protonated only once (at Fe_d_) in ODT and PDT variants. In this case, proton transfer occurs directly between the protein environment and Fe_d_. Future research on ODT or PDT variants could identify [FeFe] hydrogenase scaffolds that better optimise the active site’s catalytic performance. For example, *Tm*HydS is known to stabilise unprotonated reduced H-cluster states and may therefore serve as a suitable candidate for maturation with ODT. Understanding the interplay between the structural, electronic, and catalytic changes induced by modified active-site cofactors like ODT and PDT, as well as how different protein scaffolds influence this process, could provide valuable insights for designing enhanced semi-synthetic hydrogenases and bioinspired catalysts.

## Supplementary Information

Below is the link to the electronic supplementary material.

Supplementary Material 1

## Data Availability

Data are available in the Supplementary Information or upon request from the authors.
