# Correction: [FeFe] hydrogenases with modified azadithiolate bridgeheads serve as unidirectional hydrogen oxidation catalysts

**DOI:** 10.1007/s00775-026-02163-y

**Published:** 2026-07-31

**Authors:** Manon T. Lachmann, James A. Birrell, Simone Morra, Patricia Rodríguez-Maciá

**Affiliations:** 1 School of Chemistry and Leicester Institute for Structural and Chemical Biology, University of Leicester, University Road, Leicester, LE1 7RH UK; 2 School of Life Sciences, University of Essex, Wivenhoe Park, Colchester, CO4 3SQ UK; 3 Faculty of Engineering, University of Nottingham, Coates Building, University Park, Nottingham, NG7 2RD UK

**Correction to: JBIC Journal of Biological Inorganic Chemistry (2026) 31:131–144**

**https://doi.org/10.1007/s00775-026-02141-4**

In this article,the Figure 5 has been changed for the proposed corrections, for completeness and transparency the incorrect and correct figure has been mentioned below:

Incorrect Fig. 5

**Fig. 5:**
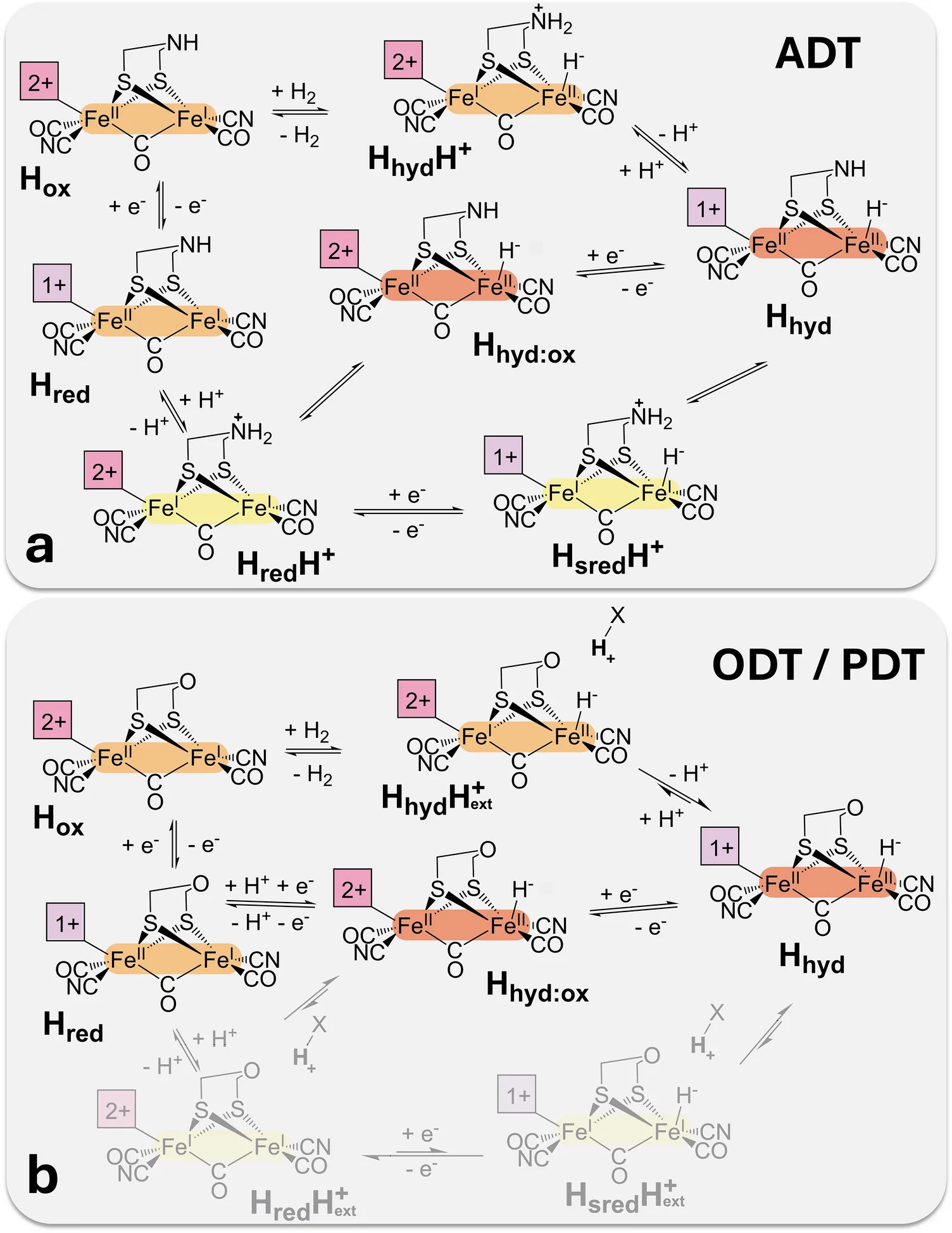
Simplified representations of the H-cluster showing proposed H_2_ oxidation pathways for ADT (**a**) and ODT and PDT (**b**). In (**b**), X denotes the species responsible for proton transfer between Fe_d_ and the protein environment; H_ext_ denotes a protonation event external to the H-cluster. Different colours represent the various oxidation states of [4Fe-4 S]_H_ and [2Fe]_H_. PCET steps are: H_red_ *⇌* H_red_H^+^ and H_hyd_H^+^ *⇌* H_hyd_

Correct Fig. 5

**Fig. 5:**
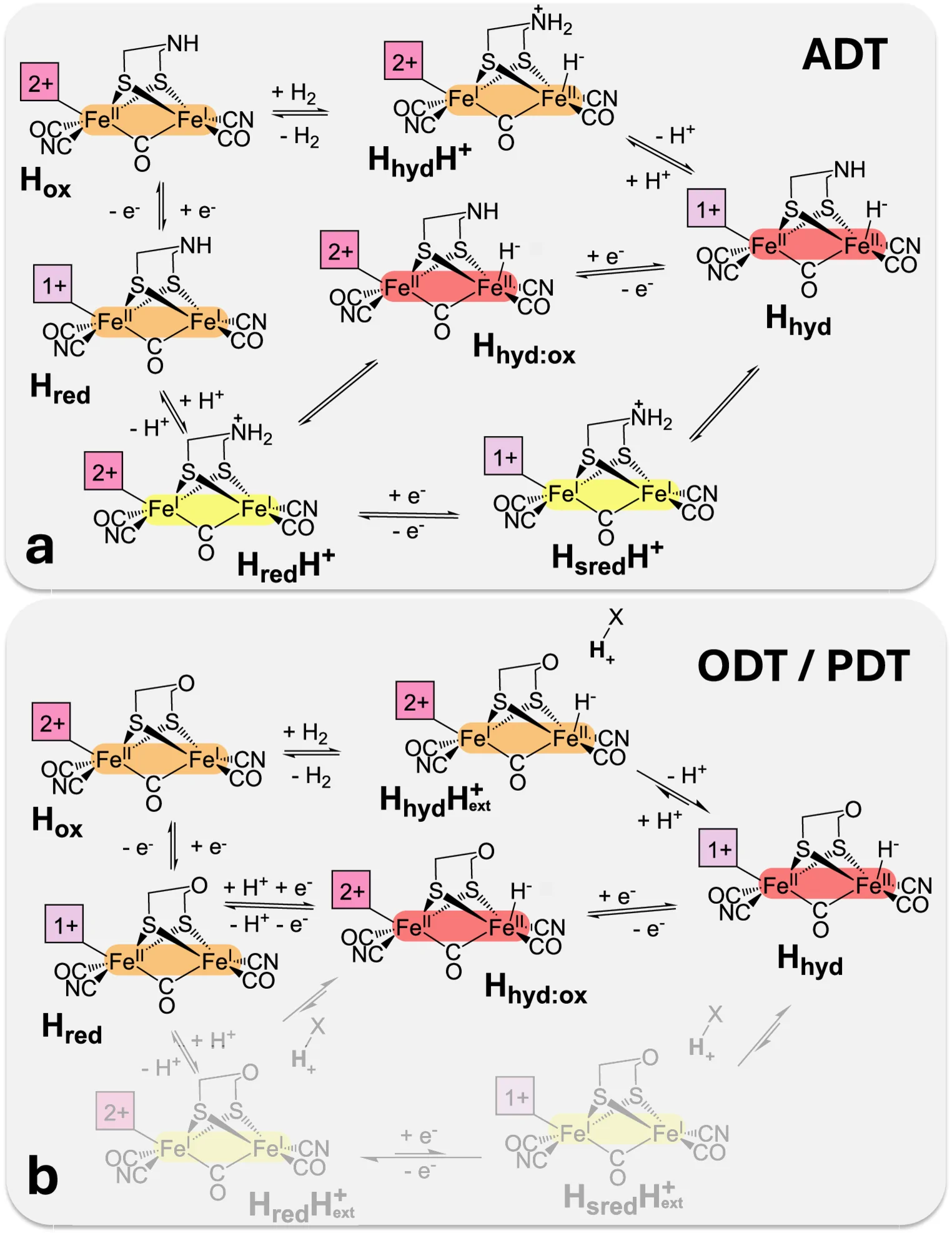
Simplified representations of the H-cluster showing proposed H_2_ oxidation pathways for ADT (**a**) and ODT and PDT (**b**). In (**b**), X denotes the species responsible for proton transfer between Fe_d_ and the protein environment; H_ext_ denotes a protonation event external to the H-cluster. Different colours represent the various oxidation states of [4Fe-4 S]_H_ and [2Fe]_H_. PCET steps are: H_red_ *⇌* H_red_H^+^ and H_hyd_H^+^ *⇌* H_hyd_

The original article has been corrected

