# Supplementary material for: [FeFe] hydrogenases with modified azadithiolate bridgeheads serve as unidirectional hydrogen oxidation catalysts
Source: J Biol Inorg Chem. 2026 Apr 4;31(3):131–44. doi: 10.1007/s00775-026-02141-4 (PMC13287228; doi:10.1007/s00775-026-02141-4)
Supplement: Supplementary file 1 — Supplementary Material 1 [file 775_2026_2141_MOESM1_ESM.pdf]

Supporting Information:

[FeFe] Hydrogenases with Modified Azadithiolate Bridgeheads serve as Unidirectional Hydrogen Oxidation Catalysts

Manon T. Lachmann[1], James A. Birrell[2], Simone Morra[3], and Patricia Rodríguez-Maciá[1*]

[1*]School of Chemistry and Leicester Institute for Structural and Chemical Biology, University of Leicester, University Road, Leicester, LE1 7RH, UK
[2] School of Life Sciences, University of Essex, Wivenhoe Park, Colchester, CO4 3SQ, UK.
[3] University of Nottingham, Faculty of Engineering, Coates Building, University Park, Nottingham, NG7 2RD, UK

*Corresponding author(s). E-mail(s): prm28@leicester.ac.uk;
Contributing authors: mtl13@leicester.ac.uk; james.birrell@essex.ac.uk; simone.morra@nottingham.ac.uk

Table of Contents

Abbreviations……………………………………………………………………………………………………3
Materials and Methods…………………………………………………………………………………………3
Supplementary Tables……………………………………………………………………………………………6
Table S1 Details of all EPR-active and EPR-silent redox states *Dd*HydAB…………..6
Supplementary Figures…………………………………………………………………………………………6
Fig. S1 Bar chart representation of H_2 oxidation TOFs……………………………………..6
Fig. S2 Ar-flushed Protein Film Electrochemistry……………………………………………7
Fig. S3 H_2-fushed Protein Film Electrochemistry……………………………………………8
Fig. S4 EPR of as is *Cr*HydA1^{ODT}………………………………………………………………9
Fig. S5 FTIR spectra after NaDT-maturation…………………………………………………10
Fig. S6 EPR of as is *Dd*HydAB^{ODT}………………………………………………………………..10
Fig. S7 Absorbance vs time plots from H_2 oxidation solution assays………………..11
References……………………………………………………………………………………………………11

Abbreviations

ADT	2-azapropane 1,3-dithiolate
As is	As-isolated
BV	Benzyl viologen
CV	Cyclic voltammogram
EPR	Electron paramagnetic resonance
Eu(II)-DTPA	Eu(II)-diethylenetriamine pentaacetate
FTIR	Fourier transform infrared
HAR	Hexaamineruthenium(III) chloride
HPI	High potential inactivation
IR	Infrared
MCT	Mercury cadmium telluride
MV	Methyl viologen
NaDT	Sodium dithionite
ODT	2-oxapropane 1,3-dithiolate
PDT	Propane 1,3-dithiolate
PCET	Proton-coupled electron transfer
PFE	Protein film electrochemistry
PGE	Pyrolytic graphite electrode
SCE	Saturated calomel electrode
SHE	Standard hydrogen electrode
TOF	Turnover frequency

Materials and Methods

Sample preparation:

*Cr*HydA1 and *Dd*HydAB apo-enzymes were produced and purified, and *Dd*HydAB was maturated with $[Fe_2(x)(CO)_4(CN)_2](NEt_4)_2$ (x = adt, odt or pdt) using reported protocols [1, 2]. $[Fe_2(x)(CO)_4(CN)_2](NEt_4)_2$ cofactors were synthesised following previously published procedures [3, 4]. *Cr*HydA1 maturation followed a procedure based on that reported by Berggren and colleagues with some modifications [5]. Apo *Cr*HydA1 was diluted to 0.2 mM in 100 mM Tris-HCl, 150 mM NaCl, pH 8. $[Fe_2(x)(CO)_4(CN)_2](NEt_4)_2$ was added to 0.6 mM from a 50 mM stock solution in DMSO, and the mixture was incubated at room temperature under a 3% H_2/97% N_2 atmosphere (Coy) for 2 h in the absence of chemical reductant. The reconstituted hydrogenase was separated from excess $[Fe_2(x)(CO)_4(CN)_2](NEt_4)_2$ using a 10 mL PD-10 column (Cytiva) equilibrated with 20 mM Tris, 30 mM NaCl, pH 8. The hydrogenase was then concentrated using 30 kDa MWCO centrifugal concentrators to 2 mM and stored anaerobically at -80°C.

Solution activity assays:

H_2 oxidation was measured by following the reduction of 1 mM benzyl viologen (BV) by enzyme in buffer mix (15 mM MES, 15 mM HEPES, 15 mM TAPS, 15 mM CHES, 15 mM

NaOAc, 100 mM NaCl), pH 7, saturated with 100% H_2 gas at 25 °C. A small amount (~5 µM) of sodium dithionite (NaDT) was added to the reaction buffer before beginning the assay to remove traces of oxygen. Measurements were performed in 1 mL plastic cuvettes using a Cole-Parmer SP-600-UV Scanning Spectrophotometer. Absorbance of $BV^{+\cdot}$ was followed at 600 nm (ε_{600} = 7.0 mM^{-1} cm^{-1}) for 3 min, and initial rates were calculated by measuring the gradient over the first 30 s. Fig. S7a provides an example of the absorbance vs time plots obtained from *Dd*HydAB^{ADT} with a control (Fig. S7b). Final values were averaged from three measurements.

H_2 production assays were performed at 37 °C in buffer mix pH 7. H_2 evolution activity was measured using gas chromatography with 10 mM reduced methyl viologen (MV) as an artificial electron donor. Reactions were prepared in 20 mL sealed headspace vials, each filled with 2 mL reaction mixture. Anaerobic conditions were achieved by flushing with pure argon for 20 min. MV was reduced through anaerobic addition of 20 mM NaDT. The reaction was initiated by anaerobic addition of the purified enzyme. Gas samples were taken using a SampleLock Gastight syringe (Hamilton) and analysed by gas chromatography. The gas chromatographer (Agilent Technologies 7820A) was fitted with a purged packed inlet, a Carboxen-1010 PLOT column (30 m × 0.53 mm I.D.), and a thermal conductivity detector; argon served as the carrier gas. Values are averaged from three measurements.

Protein film electrochemistry (PFE):

A gas-tight, water-jacketed three-electrode electrochemical cell was used in an anaerobic glovebox (Glovebox Technology Ltd. UK) for experiments, with H_2 gas flow controlled using a mass flow controller (Sierra Instruments). Electrochemical control was achieved using an Autolab potentiostat (PGSTAT128N) controlled by Nova software. The three-electrode system consisted of a saturated calomel electrode (SCE) as the reference electrode placed in 0.1 M NaCl solution in a separate arm and connected to the main cell via a luggin capillary. The main cell contained a platinum wire counter electrode and pyrolytic graphite electrode (PGE) as the working electrode with an area of 0.03 cm^2, which was rotated at 3300 rpm to avoid mass-transport limitations. Before adsorption, the PGE was polished inside the glovebox with P400 sandpaper (3M). 3 µL enzyme (6 µM in 10 mM MES buffer, pH 5.8) was deposited on the electrode surface, allowed to adsorb for 5 min, and then gently rinsed to remove any unbound protein before inserting the electrode into the electrochemical cell. All potentials were measured in buffer mix, pH 6 and 7, at 25 °C against the SCE and converted to values versus the standard hydrogen electrode (SHE), which was determined after calibration of the reference electrode using the standard ferrocenemethanol (FcMeOH/$FcMeOH^+$) redox couple [6].

Infrared (IR) spectroscopy:

IR samples were prepared in an anaerobic glovebox (Belle Technology UK Ltd.) under an N_2 atmosphere by mixing 4 µL of enzyme in 20 mM Tris-HCl, 30 mM NaCl buffer pH 8 with either an oxidant or a reductant; hexaamineruthenium(III) chloride (HAR, oxidant) and sodium dithionite (NaDT, reductant) were added in 2-10x excess from 100 mM stocks in 100 mM Tris-HCl, 150 mM NaCl buffer pH 8. Eu(II)-diethylenetriamine pentaacetate (Eu(II)-DTPA, reductant) was prepared by dissolving $EuCl_2$ in 0.5 M HCl to a 100 mM concentration and diethylenetriamine pentaacetic acid (DTPA) in 0.5 M NaOH to 100 mM. The two solutions were

then mixed in a 1:1 ratio to produce a 50 mM Eu(II)-DTPA solution. pH indicator paper was used to ensure the pH was close to neutral, and the solution was added to the enzyme in a 10x excess. As-isolated (as is) samples were left untreated after maturation. For H_2-flushed samples, 5 µL sample was aliquoted into an open 200 µL Eppendorf tube, which was hermetically sealed in a glass vial and flushed with 100% H_2 for 10 minutes. The samples were then sandwiched between two CaF_2 windows (Crystan) with a 25 µm Teflon spacer (PIKE technologies) and sealed inside a commercial IR cell (PIKE technologies). IR spectra were collected using a Bruker VERTEX 80 Fourier transform infrared (FTIR) spectrometer equipped with a mercury cadmium telluride (MCT) detector, which was cooled with liquid nitrogen. Spectra were recorded using OPUS software with a resolution of 2 cm^{-1}, aperture setting of 1.5 mm, and a scan velocity of 20 kHz with an average of 1024 scans. Once recorded, water vapour subtraction and data cutting were completed in OPUS, followed by baseline subtraction using Kazan Viewer in the MATLAB environment. Figures were prepared using Origin.

Electron paramagnetic resonance (EPR) spectroscopy:

EPR samples were prepared in an anaerobic glovebox (Coy) under a 3% H_2/97% N_2 atmosphere. Protein samples were diluted to 0.2 mM, and a 2-10x excess of oxidant or reductant was added (prepared as described above) to make up a total volume of 200 µL. Samples were frozen in liquid nitrogen and measured at 30 K and 0.1 mW power on a Bruker E500 X-band (9.36 GHz) EPR spectrometer. Sample temperature was maintained using liquid helium with an Oxford Instruments transfer line and an ITC temperature controller. EPR spectra were collected with 100 kHz modulation frequency, 10 Gauss modulation amplitude, and 80 ms time constant. EPR spectra were background subtracted using Kazan Viewer in the MATLAB environment, and EPR spectral simulations were performed using the esfit function in EasySpin, also in MATLAB.

Supplementary Tables

Table S1 Details of all EPR-active and EPR-silent redox states of *Dd*HydAB

Redox state	Distal F-cluster (Fd)	Proximal F-cluster (Fp)	H-cluster (H)	Spin coupling between	Spin(s)/mole
H_{ox}	Ox	Ox	**Ox**	-	1
Fd_{red}	**Red**	Ox	**Ox**	-	2
$Fd_{red}Fp_{red}$	**Red**	**Red**	**Ox**	Fd, Fp, H	3
$Fd_{red}H_{red}$	**Red**	Ox	Red	-	1
Fp_{red}	Ox	**Red**	**Ox**	Fp and H	2
$Fp_{red}H_{red}$	Ox	**Red**	Red	-	1
$Fd_{red}Fp_{red}H_{red}$	**Red**	**Red**	Red	Fd, Fp	2

The three redox-active clusters in *Dd*HydAB (Fd, Fp, and H) are denoted 'ox' or 'red', meaning oxidised or reduced. Although the H-cluster comprises $[4Fe\text{-}4S]_H$ and $[2Fe]_H$, these are spin-coupled and act as one EPR-active centre. The bold redox states symbolise an EPR-active cluster.

Supplementary Figures

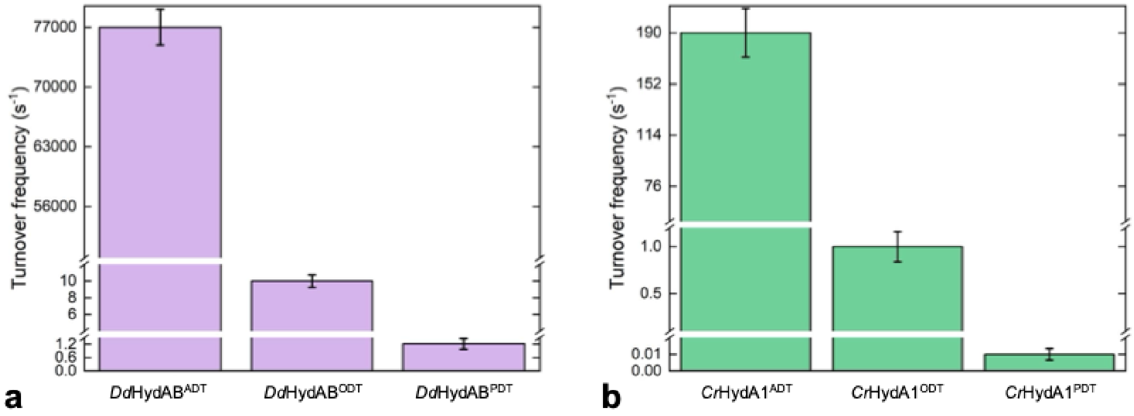

Fig S1. H_2 oxidation TOFs of different cofactors in *Dd*HydAB (**a**) and *Cr*HydA1 (**b**) measured through the reduction of 1 mM benzyl viologen (BV) by hydrogenase in buffer mix pH 7 at 25 °C

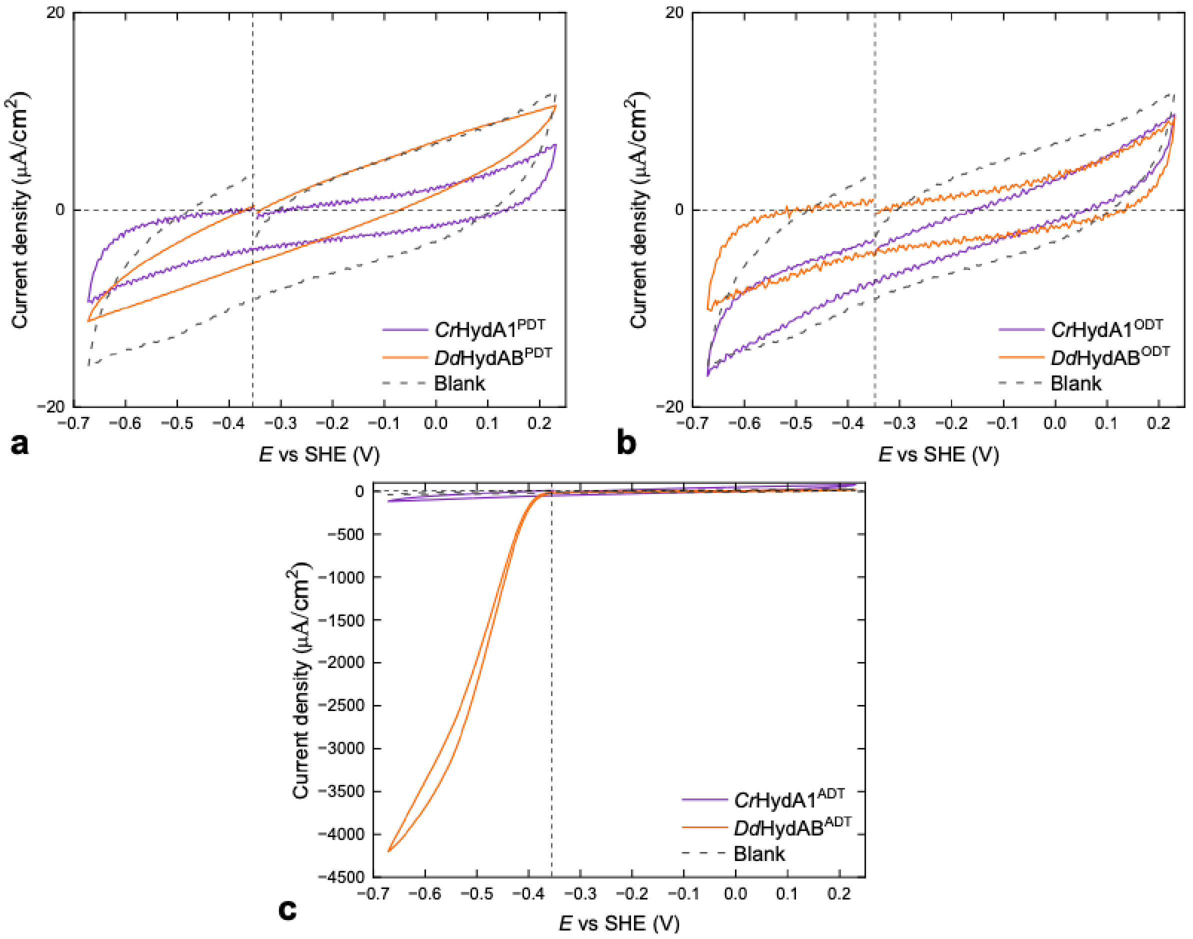

Fig. S2 CVs of PDT (**a**), ODT (**b**) and ADT (**c**) maturated in both *Cr*HydA1 (purple) and *Dd*HydAB (orange) performed in buffer mix pH 6, 0.1 V s^{-1} scan rate, 3300 rpm and 1000 mL min^{-1} Ar. The electrode blank (grey dashed line) is displayed. The vertical dashed line corresponds to $E(2H^+/H_2)$ (which at pH 6 is -0.355 V), and the dashed horizontal line corresponds to the zero current density

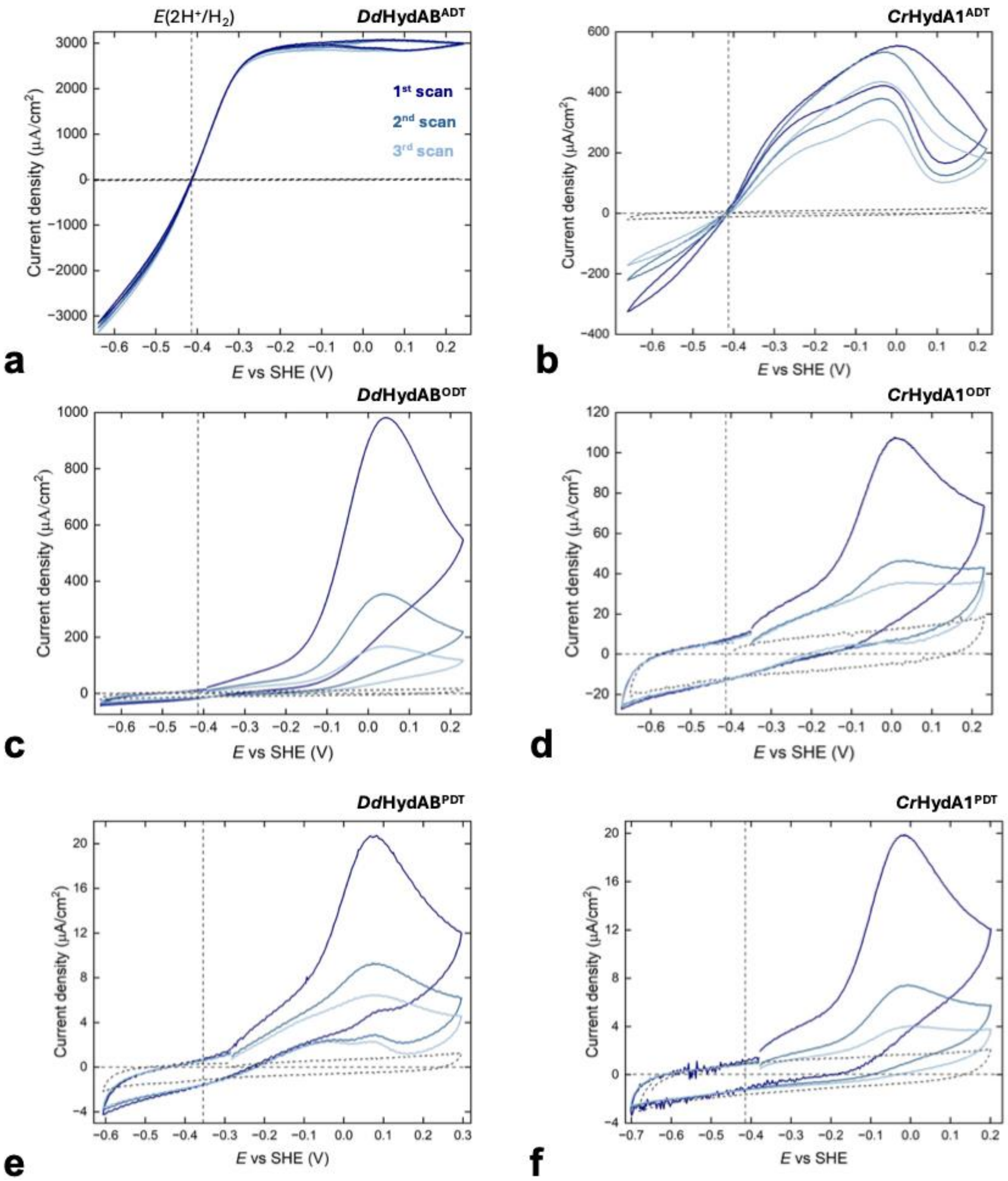

Fig. S3 Cyclic Voltammograms (CVs) of *Dd*HydAB^{ADT} (**a**), *Cr*HydA1^{ADT} (**b**), *Dd*HydAB^{ODT} (**c**), *Cr*HydA1^{ODT} (**d**), *Dd*HydAB^{PDT} (**e**), and *Cr*HydA1^{PDT} (**f**). The first, second and third recorded CVs are coloured accordingly as shown in a. The electrode blank (grey dashed line) is displayed. The vertical dashed line corresponds to $E(2H^+/H_2)$ (which at pH 7 is -0.413 V vs SHE and at pH 6 is -0.355 V), and the dashed horizontal line corresponds to the zero current density. Measurements were performed in buffer mix pH 6 (e) or pH 7 (a-d, f), 0.1 V s^{-1} scan rate, 3300 rpm, 1000 mL min^{-1} H_2 and at 25 ^{o}C

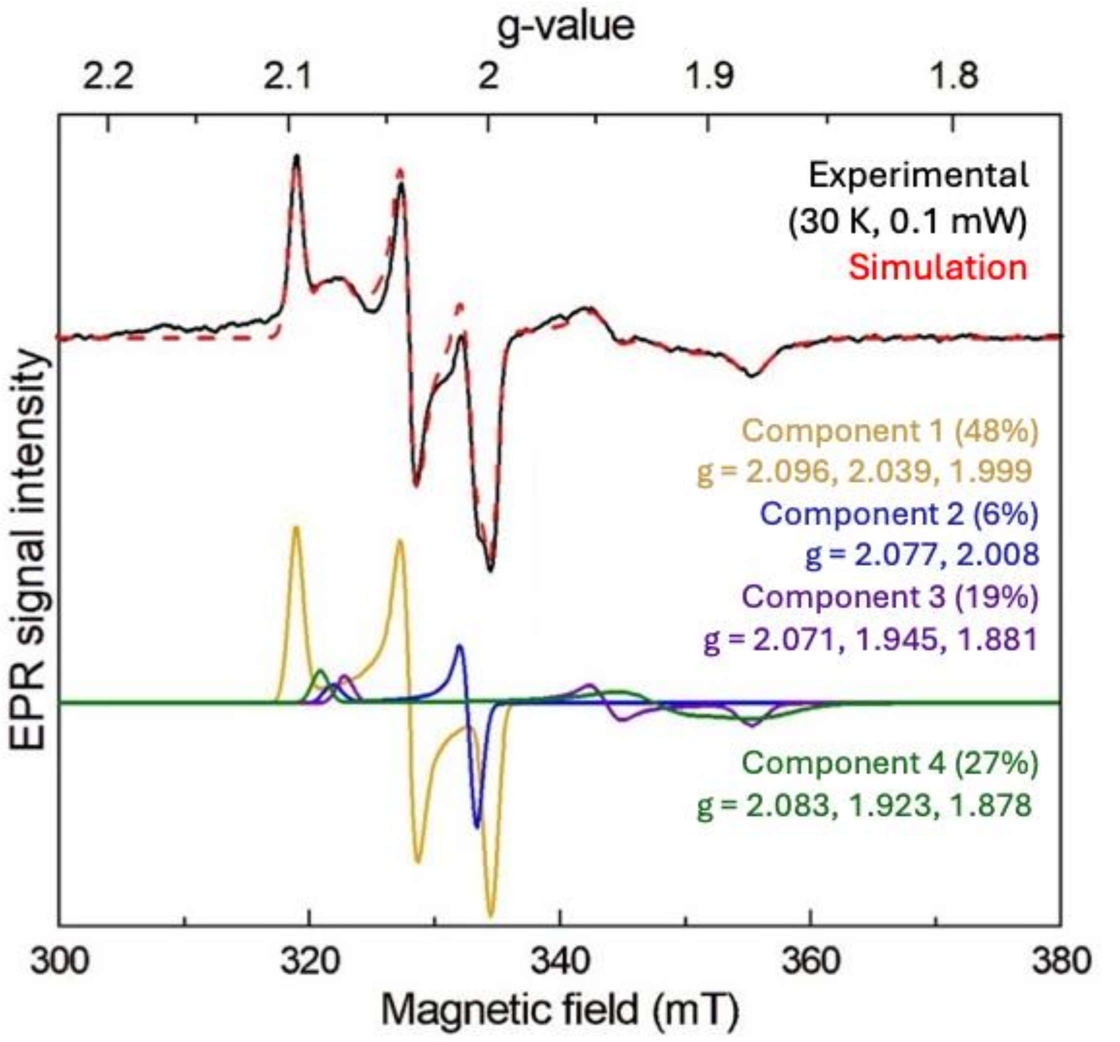

Fig. S4 EPR spectrum of *Cr*HydA1^{ODT} as isolated. The experimental (black) and simulated (red dashed) spectra are overlaid. The simulated spectrum is separated into its different components below. The percentages alongside the components represent their contributions to the sample based on the relative numbers of spins calculated from the double integrals of the spectra

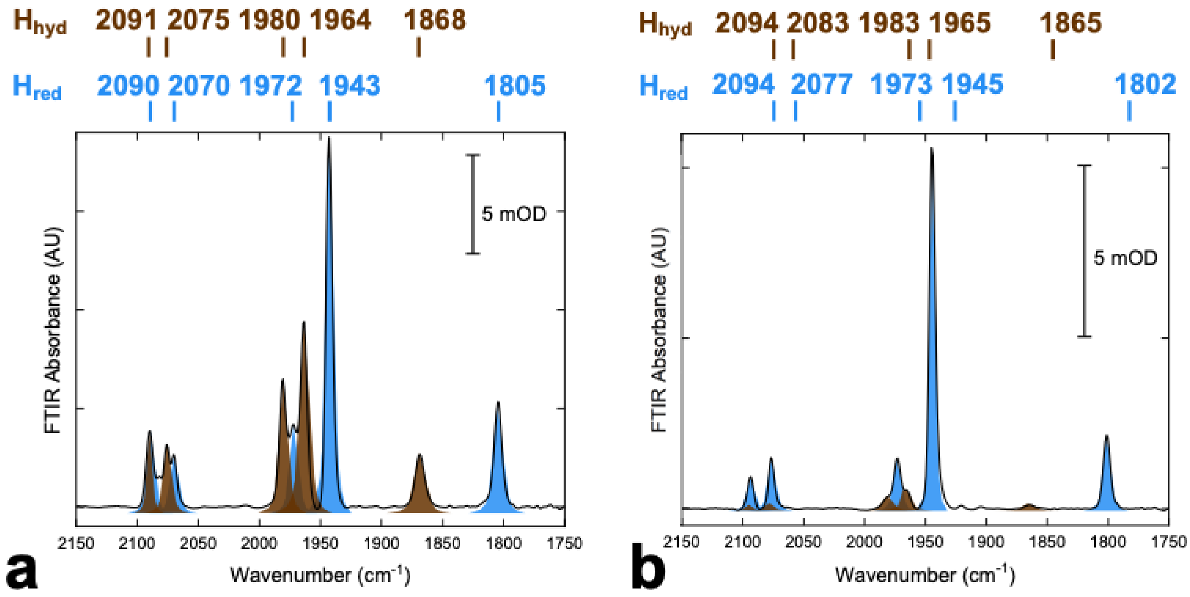

Fig. S5 FTIR spectra of *Cr*HydA1^{ODT} (**a**) and *Dd*HydAB^{ODT} (**b**) after maturation with 10 mM NaDT

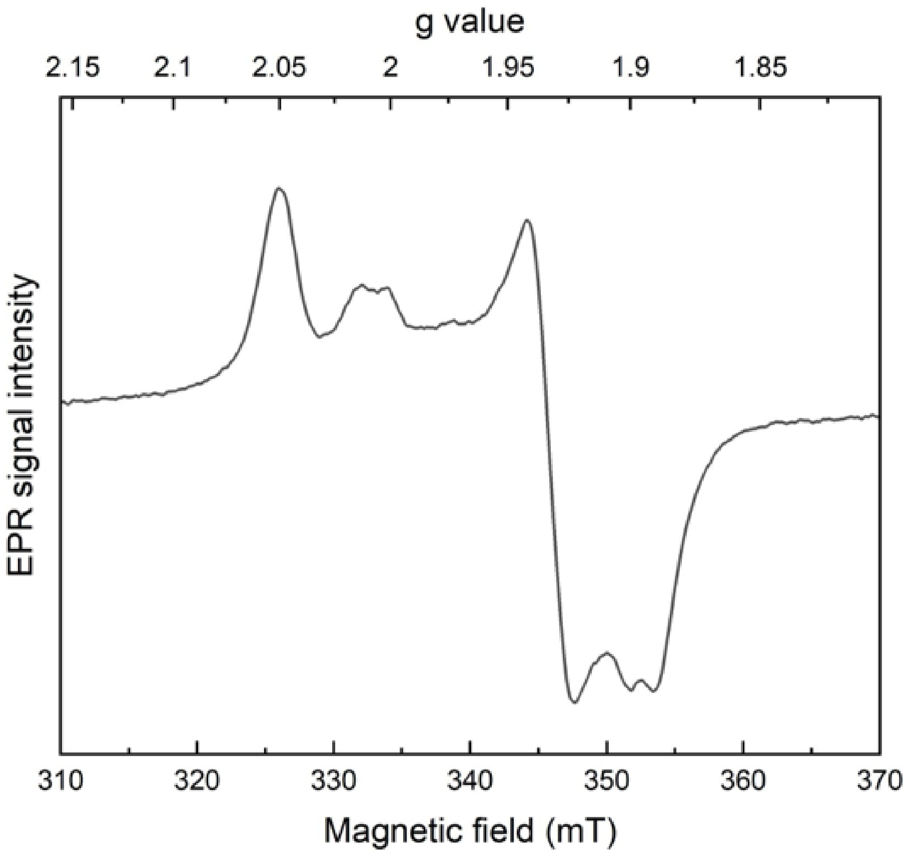

Fig. S6 EPR spectrum of as is *Dd*HydAB^{ODT}

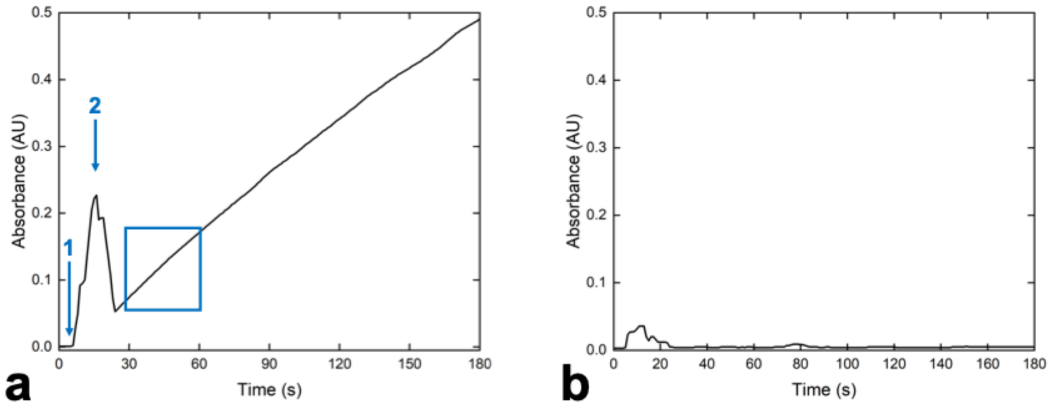

Fig. S7a Example of absorbance vs time plot generated from H_2 oxidation solution activity assays performed on *Dd*HydAB^{ADT}at 2.8x10^{-9} mM. Absorbance was measured at 600 nm for 3 min. **1** corresponds to the addition of hydrogenase (in this case, *Dd*HydAB^{ADT}), and **2** corresponds to pipette mixing. Rates were calculated from the gradient over the first 30 s of the reaction after mixing. **b** shows the control; the same procedure was followed, but the BV solution was spiked with buffer only

References

1. Birrell JA, Wrede K, Pawlak K, Rodriguez-Maciá P, Reijerse EJ, Rüdiger O, Lubitz W (2016) Artificial Maturation of the Highly Active Heterodimeric [FeFe] Hydrogenase from *Desulfovibrio desulfuricans* ATCC 7757. Isr. J. Chem. https://doi.org/10.1002/ijch.201600035
2. Kuchenreuther JM, Grady-Smith CS, Bingham AS, George SJ, Cramer SP, Swartz JR (2010) High-Yield Expression of Heterologous [FeFe] Hydrogenases in *Escherichia coli*. PLoS One https://doi.org/10.1371/journal.pone.0015491
3. Siebel JF, Adamska-Venkatesh A, Weber K, Rumpel S, Reijerse E, Lubitz W (2015) Hybrid [FeFe]-hydrogenases with modified active sites show remarkable residual enzymatic activity. Biochem. https://doi.org/10.1021/bi501391d
4. Barton BE, Olsen MT, Rauchfuss TB (2008) Aza- and Oxadithiolates Are Probable Proton Relays in Functional Models for the [FeFe]-Hydrogenases. J. Am. Chem. Soc. https://doi.org/10.1021/ja8057666
5. Berggren G, Adamska A, Lambertz C, Simmons TR, Esselborn J, Atta M, Gambarelli S, Mouesca JM, Reijerse E, Lubitz W, Happe T, Artero V, Fontecave M (2013) Biomimetic assembly and activation of [FeFe]-hydrogenases. Nature https://doi.org/10.1038/nature12239
6. Rodriguez-Maciá P, Dutta A, Lubitz W, Shaw WJ, Rüdiger O (2015) Direct Comparison of the Performance of a Bio-inspired Synthetic Nickel Catalyst and a [NiFe]-Hydrogenase, Both Covalently Attached to Electrodes. Angew. Chem. Int. Ed. https://doi.org/10.1002/anie.201502364
